# Individual and community-level factors associated with repeat induced abortion among women in Ghana: a multivariable complex sample logistic regression analysis of 2017 Ghana maternal health survey

**DOI:** 10.1186/s12889-024-18948-2

**Published:** 2024-05-28

**Authors:** Isaac Yeboah, Joshua Okyere, Desmond Klu, Pascal Agbadi, Martin Wiredu Agyekum

**Affiliations:** 1 Institute of Work, Employment and Society, University of Professional Studies, Accra, Ghana; 2 Department of Population and Health, University of Cape Coast, Cape Coast, Ghana; 3 Institute of Heath Research (IHR), University of Health and Allied Sciences, Ho, Ghana; 4 Department of Sociology and Social Policy, Lingnan University, SAR, Hong Kong, China; 5 Institute for Educational Research and Innovation Studies (IERIS), University of Education, Winneba, Ghana

**Keywords:** Repeat, Induced abortion, Complex sample analysis, Ghana

## Abstract

**Background:**

Repeat induced abortion is a serious public health issue that has been linked to adverse maternal health outcomes. However, knowledge about repeat induced abortion and its associated factors among reproductive age women in Ghana is very scarce. The objective of this study is to examine individual and community factors associated with repeat induced abortion in Ghana which would be helpful to design appropriate programmes and policies targeted at improving the sexual and reproductive health of women.

**Methods:**

We used secondary cross-sectional data from the 2017 Ghana Maternal Health Survey. The study included a weighted sample of 4917 women aged 15–49 years with a history of induced abortion. A multivariable complex sample logistic regression analysis was used to investigate individual and community factors associated with repeat induced abortion among women in Ghana. Adjusted odds ratios (AOR) with 95% confidence intervals (CI) was used to measure the association of variables.

**Results:**

Of the 4917 reproductive women with a history of abortion, 34.7% have repeat induced abortion. We find that, compared to women who experience single induced abortion, women who experience repeat abortion are age 25–34 years (AOR:2.16;95%CI = 1.66–2.79) or 35–49 years (AOR:2.95;95%CI:2.18–3.99), have Middle/JHS education (AOR:1.69;95%CI = 1.25–12.27), use contraceptive at the time of conception (AOR:1.48: 95%CI = 1.03–2.14), had sexual debut before 18 years (AOR:1.57; 95%CI: 1.33–1.85) and reside in urban areas (AOR:1.29;95%CI = 1.07–1.57). On the other hand, women who reside in Central (AOR:0.68;95%CI: 0.49–0.93), Northern (AOR:0.46;95%CI:0.24–0.88), Upper West (AOR:0.24; 95%CI: 0.12–0.50) and Upper East (AOR:0.49; 95%CI = 0.24–0.99) regions were less likely to have repeat induced abortion.

**Conclusion:**

The study showed that both individual and community level determinants were significantly associated with repeat induced abortion. Based on the findings, it is recommended to promote sexual and reproductive health education and more emphasis should be given to adult, those with early sexual debut, those with Middle/JHS education and those who live in urban centers.

## Background

Unwanted pregnancies continue to be one of the most common public health issues worldwide [1]. According to a Lancet report, nearly 121 million unintended pregnancies were reported worldwide between 2015 and 2019, with sub-Saharan Africa (SSA) countries accounting for 87% of these pregnancies [2]. In all cases of unintended pregnancies, the woman is faced with the decision of whether to keep or abort the pregnancy. However, available data show that between 2015 and 2019, 73 million abortions were performed on women of reproductive age each year [3]. Between 2015 and 2019, the abortion rate in SSA was 33 per 1,000 women of reproductive age [3]. According to a study conducted in Ghana, approximately 25% of pregnancies among women aged 15 to 49 years are terminated [4]. These figures indicate a serious public health concern in Ghana, with some women undergoing induced abortion procedures more than once, a practice known as repeat induced abortion in the literature [1, 5–8].

Clinically, the term repeat induced abortion refers to having more than one induced abortion before the 28th week of pregnancy [9]. The prevalence of repeat abortions varies greatly across the globe. For example, an Italian study reported a 60.6% prevalence of repeat abortions [10]. In the Netherlands, the prevalence of repeat abortion is 36% [11]; Georgia, Kenya and Nigeria report prevalence rates of 69.9%, 14.3% and 23%, respectively [12–14]. Repeat abortions have been linked to adverse maternal health outcomes, such as ectopic pregnancy, placenta previa, infertility, preterm birth, and the possibility of developing life-threatening problems such as hemorrhage, anemia, and sepsis [15, 16]. As a result, evidence-based research is required to understand the factors that influence women to have a repeat induced abortion.

Previous research in the United States [17], Kenya [13], and Vietnam [18] have found that demographic, socioeconomic, and contextual factors are significantly associated with repeat induced abortions among women of reproductive age. In addition to these factors is legal environment on abortion. In Ghana, abortion is legal only when a woman’s life is in danger, when the procedure would protect her physical and mental health, and when there is rape or incest [19]. As a result of the legal context surrounding induced abortion in Ghana, it is critical to conduct research to better understand the factors associated with repeat induced abortion in the country. To the best of our knowledge, no study in Ghana has used a nationally representative dataset to investigate the factors associated with repeat abortion. This situation presents a serious problem because there is limited empirical evidence to inform policy and intervention to reduce the incidence of repeat induced abortions. Moreover, it signifies a substantial knowledge gap in the current scholarship and understanding of the factors that are associated with repeat induced abortion.

Despite studies on repeat induced abortion in different parts of Ghana, most of them focused on individual level factors using basic logistic regression model covering small sample size [8, 9]. The behaviour of individual towards repeat induced abortion are not only attributed by individual factors but also by community level factors since the individual woman is nested within the communities. In the Ghana Maternal Health Survey (GMHS) data, individuals are nested within a cluster/community, and their characteristics may be similar to those living in the same cluster/community compared to the rest of Ghana. Adjusting for the hierarchical nature of the data provides more realistic standard errors and estimates, as complex sample regression analysis will explicitly model the correlation of responses at the group-level. This study aimed to identify individual level and community level factors associated with repeat induced abortion among women with history of induced abortion using multilevel logistic regression analysis.

## Theoretical framework

This study is underpinned by the social learning theory and rationale choice theory. The social learning theory is one of the learning theories that see the environment as the major force in any human behaviour [20]. According to proponents of social learning theory, human behaviour is influenced by observation, modeling and imitations [21]. Individuals learn from interactions with others in a social context by observing the behaviours of others. That is, an individual learns behavior by observing others in a social milieu (including as family, friends, teachers, neigbours and church groups). After observing others, individuals tend to assimilate and imitate that behaviour, especially when their observations involve associated rewards or positive experiences. Bandura asserts that reinforcement can account for social learning. Reinforcement is when an individual is aware of prior experience consequences. Lastly, imitation is when individuals engage in behaviours witnessed from others [21]. If the consequence to abortion is positive there is the likelihood of its occurrence and vice versa. The rationale choice theory proposed by Ronald V. Clarke and Derek B. Cornish in 1986 argues that people are rationale human beings and behave the way they do because they belief performing a chosen action has more benefits than costs [22]. They further argue that the individual uses a decision-making process whereby the positive and negative aspects of taking a particular action is weighted. If the individual perceives that there are more reasons for proceeding with an action irrespective of the existing negative share of the decision, at the very least, an attempt will be made [23]. Following this theory, a woman will take the decision to abort a baby if the decision will benefit her.

Using social learning and rationale choice theories, the study hypothesized that individual and community level factors act as factors for women in Ghana’s exposure to repeat induced abortion. We also hypothesized that the risk of repeat induced abortion would differ by region and place of residence (rural/urban settings) due to difference in cultural values and norms surrounding abortion.

## Methods

### Study setting

This study was done in Ghana, which is located in the Western part of Africa. The country is labelled as agrarian and city-based population. It has a total population of 30,832,019 where 15, 610,149 are females representing 50.7% [24]. The country has an average national household size of 3.6 persons. Majority of the population (56.7%) reside in urban areas [24]. The county has fertility rate of 3.1, infant mortality rate of 28 deaths per 1000 births and under-5 mortality rate of 40 deaths per 1000 children [25].

### Data source, sample and design

We relied on the individual file (women data) of the 2017 Ghana Maternal Health Survey (GMHS). The GMHS is a retrospective nationally representative cross-sectional survey that collects data for monitoring maternal health in Ghana. The 2017 GMHS data were collected from June 2017 to October 2017, implemented by the Ghana Health Service (GHS) and Ghana Statistical Service (GSS) and received technical support from the ICF. For the 2017 GMHS, 27,001 households were selected for the sample, of which 26,500 were occupied at the time of fieldwork. The surveyed women were sampled using a multi-stage cluster sampling approach. A total of 900 enumeration areas (EAs) were randomly selected across the nation and households in these EAs were then systematically selected [26]. Secondly, a household listing was carried out in selected EAs to serve as a sampling frame for the selection of households in the second stage. The interviewed households were 26, 324, resulting in a response rate of 99%. The detailed sampling procedure, sample size, and findings are available in the national report [26]. Written informed consent was obtained during the data collection process for all participants with reproductive ages of 15–49 years. The women data consist of 25,062 women of reproductive age (15–49 years). We restricted our sample to women who had ever experienced induced abortion, as these women had the chance of having repeat abortion. The exclusion criteria were those who met the inclusion criteria but had missing data. This led to a weighted sample size of 4917 that was finally used for the analysis.

### Measures

#### Outcome variable

The outcome variable was ‘repeat induced abortion’ measured in the GMHS by the item ‘How many pregnancies have ended this way (abortion) in your lifetime?’. The outcome variable yielded two different categories: single abortion and repeat abortion. Single abortion denotes women who have had only one induced abortion in their lifetime while repeat abortion refers to women who have had more than one induced abortion in their lifetime.

### Independent variables

To accommodate the hierarchical nature of the GMHS data, two level (individual and community) of factors with potential effects on repeat induced abortion were considered for analysis.

### Individual-level factors

The individual-level factors were age (coded as 1 = 15–24, 2 = 25–34, 3 = 35–49), educational attainment (coded as 1 = no education, 2 = primary education, 3 = Middle/JHS, 4 = secondary, 5 = higher), marital status (coded as 1 = currently married, 2 = cohabiting, 3 = not in union), contraceptive use at the time of conception (coded as 1 = yes, 2 = no), age at first sex (coded as 1 = < 18 years, 2 = 18 years above), parity (coded as 0 = no birth, 1 = 1–3, 2 = 4–5, 3 = 6+).

#### Community-level factors

Community-level variables (community media exposure, community knowledge of ovulation and community-level education) were created from individual-level variables by aggregating them at the cluster (community) level by using the bysort command. We obtained the proportion of each community-level characteristic and the values were ranked into tertiles as low, medium, and high. The low tertile (tertile 1) represent lowest score and least advantaged, tertile two indicates medium score and tertile 3 represent highest score and most advantaged. Community-level education, which was the proportion of women with higher education in the community and categorized as low, or high. The community knowledge of ovulation level was categorized into tertiles and classified as low, medium, or high knowledge of ovulation level. Lastly, community media exposure was categorized into low and medium level. Two direct community level variables were used in the analysis; place of residence and region of residence. The GHMS collects data on place of residence that describes the characteristics of clusters. Place of residence was coded as 1 = rural and 2 = urban while region of residence was coded as 1 = western, 2 = central, 3 = Greater Accra, 4 = Volta, 5 = Eastern, 6 = Ashanti, 7 = Brong Ahafo, 8 = Northern, 9 = Upper East and 10 = Upper West. Though the study used 2017 dataset which had 10 administrative regions, the results are representative of the current Ghana with 16 regions because of the sampling approach of the study. The study used a multi-stage cluster sampling where each administrative region is separated into urban and rural areas yielding sampling strata. Besides, the additional administrative regions were all part of the previous 10 administrative regions. For instance, the old Brong Ahafo region has been divided into Bono, Bono East and Ahafo regions.

### Statistical analysis

The analysis focused on repeat induced abortion, and all analyses were performed using Stata version 16.0 (StataCorp, Texas, USA). First, the background characteristics of women 15–49 years were explored. Second, a bar chart was used to show the different abortion statuses (single abortion and repeat abortion). Next, we examined the significance of difference in the prevalence of induced abortion using chi-square test. That is, cross tabulation was used to measure prevalence across covariates. Given that the GMHS is a multi-stage stratified design, a multivariable complex sampling design logistic regression analysis was used to examine the association between individual and community factors and repeat induced abortion in Ghana using the recent GMHS dataset. The complex sampling design analysis eliminates the possibility of underestimating the standard errors associated with the confidence intervals as well as regression coefficients. We applied weighting in all the analyses. First, we weighted the dataset using the commands (gen wgt = QWEIGHT/1,000,000) to obtain unbiased estimates. Additionally, the survey command in Stata Svyset [pw = wgt], psu (QHCLUST) was used to adjust for the complex sampling structure of the data in all the analysis. Afterwards, the dataset was appended and used for the analysis. The ‘svyset’ command was activated in Stata 16 to set the data in complex survey analysis. The primary sampling units, sample strata and sample weights, were all adjusted in univariate, bivariate and multivariable analysis. This helped to achieve correct estimates of confidence intervals and standard errors of predicted estimates, especially in the multivariable logistic regression model.

## Results

### Prevalence of repeat induced abortion

#### Individual characteristics

**Fig. 1:**
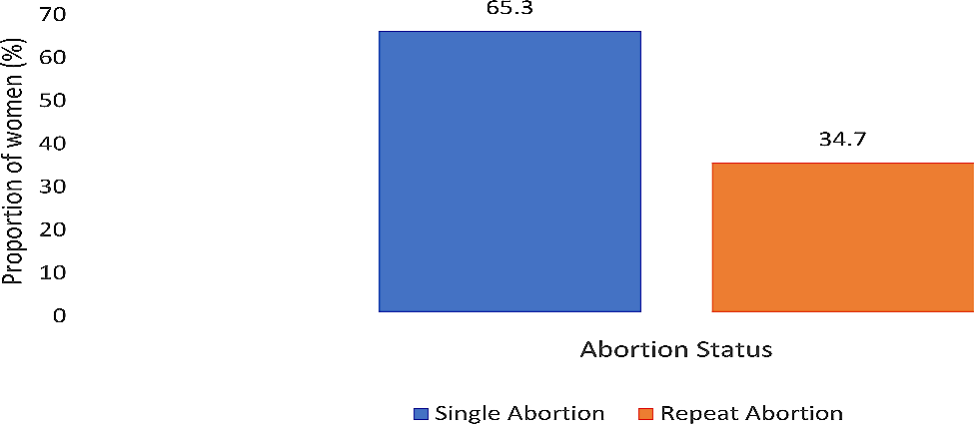
Percentage distribution of women by abortion status, 2017, Ghana

Table 1 presents the prevalence of single and repeat induced abortion by individual characteristics of the study population. In total, 4917 women were included. The overall prevalence of repeat induced abortion was 34.7% (Fig. 1). Prevalence of repeat induced abortion varied among different age groups of women, with the highest prevalence of 51.8% seen among women aged 35–49 years. The lowest prevalence (9.6%) was seen among young women aged 15–24 years. Prevalence was greatest (54.2%) among women with Middle/JHS education compared with those with higher education (5.7%). The prevalence of repeat abortion was higher (40.1%) among those currently married compared with 29.0% who were not married. The prevalence of repeat induced abortion was higher among those not using contraception at the time of conception (77.2%), those with parity of 1–3 (55.3%) and those whose sexual debut was less than 18 years (66.9%).

**Table 1:** Prevalence of single and repeat abortion by individual characteristics

Characteristics	Single abortion (%)	Repeat abortion (%)	*P* value	Weighted Sample size *n* (%)
Age				
15–24	20.8	9.6	< 0.001	831 (16.9)
25–34	40.4	38.6		1956 (39.8)
35–49	38.8	51.8		2130 (43.3)
**Educational Attainment**				
No education	11.7	9.2	< 0.001	531 (10.8)
Primary education	16.8	16.8		826 (16.8)
Middle/JHS	45.9	54.2		2398 (48.8)
Secondary	18.9	14.2		849 (17.3)
Higher	6.7	5.7		313 (6.4)
**Marital Status**				
Currently married	35.6	40.1	0.004	1830 (37.2)
Cohabiting	31.8	30.9		1548 (31.5)
Not in union	32.6	29.0		1539 (31.3)
**Contraceptive use at the time of conception**				
Yes	16.8	22.8	0.033	352 (18.7)
No	83.2	77.2		1528 (81.3)
**Age at first Sex**				
< 18 years	59.5	66.9	< 0.001	3051 (62.0)
18 years above	40.5	33.1		1866 (38.0)
**Parity**				
No birth	17.4	9.5	< 0.001	721 (14.7)
1–3	55.4	55.3		2725 (55.4)
4–5	18.6	25.5		1032 (21.0)
6+	8.5	9.7		439 (8.9)

### Community characteristics

The prevalence of repeat induced abortion (Table 2) was higher among those residing in the urban areas (70.3%) compared with the rural areas (29.7%). Prevalence of repeat induced abortion varied among women in different administrative regions in Ghana with the highest of 26.1% seen among those residing in Ashanti region. The lowest prevalence was seen among those residing in Upper East region (0.2%). The prevalence of repeat induced abortion was higher (69.6%) among those with medium community media exposure.

**Table 2:** Prevalence of single and repeat induced abortion by community characteristics

Characteristics	Single induced abortion (%)	Repeat induced abortion (%)	*P* value	Weighted Sample size *n* (%)
Place of Residence				
Rural	36.3	29.7	< 0.001	1672 (34.0)
Urban	63.7	70.3		3246 (66.0)
**Region of Residence**				
Western	13.3	14.5	0.001	673 (13.7)
Central	8.7	7.5		409 (8.3)
Greater Accra	24.7	26.0		1236 (25.1)
Volta	6.7	6.0		319 (6.5)
Eastern	10.3	10.0		501 (10.2)
Ashanti	23.3	26.1		1194 (24.3)
Brong Ahafo	10.1	8.8		475 (9.7)
Northern	1.3	0.5		50 (1.0)
Upper East	0.8	0.2		30 (0.6)
Upper West	0.8	0.3		30 (0.6)
**Community level of education**				
Low	65.9	65.1	0.713	3228 (65.6)
High	34.1	34.9		1689 (34.4)
**Community media exposure**				
Low	35.0	30.4	0.020	1643 (33.4)
Medium	65.0	69.6		3274 (66.6)
**Community ovulation knowledge**				
Low	31.7	30.3	0.770	1535 (31.2)
Medium	36.0	36.7		1782 (36.2)
High	32.3	33.0		1600 (32.5)

### Individual-and community-level determinants of repeat induced abortion

#### Individual-level factors

The strength of association was assessed using a single multivariable logistic regression model. The model adjusted for individual and community level characteristics. With the individual-level factors (Table 3), the likelihood of repeat induced abortion was higher among women aged 25–34 years (AOR:2.16; 95%CI = 1.66–2.79), those aged 35–49 years (AOR:2.95;95%CI: 2.18–3.99), those with Middle/JHS education (AOR:1.68;95%CI = 1.25–2.27), those using contraception at the time of conception (AOR:1.48;95%CI = 1.03–2.14) and women whose age at first sex was when less than 18 years old (AOR:1.57;95%CI = 1.33–1.85) compared with those aged 15–24 years, those with no education, those not using contraception at the time of conception and those whose sexual debut was at 18 years above respectively.

### Community-level factors

In terms of the community factors, women residing in urban areas (aOR:1.29;95%CI = 1.07–1.57) were more likely to have repeat induced abortion compared with those in the rural areas. Those residing in central region (aOR:0.68;95%CI = 0.49–0.93), Northern region (aOR:0.46; 95%CI = 0.24–0.88), Upper East region (aOR:0.24;95%CI = 0.12–0.50) and Upper West region (aOR:0.49;95%CI = 0.24–0.99) were less likely to have repeat abortion compared with women residing in Western Region.

**Table 3:** Multivariable complex sample logistic regression estimates of individual and community predictors of repeat induced abortion

Individual Characteristics	AOR	*P*-value	95% CI	
Age			Lower	Upper
15–24 (ref)				
25–34	2.16	**< 0.001**	1.66	2.79
35–49	2.95	**< 0.001**	2.18	3.99
Education				
No education (ref)				
Primary	1.30	0.104	0.95	1.79
Middle/JHS	1.68	**0.001**	1.25	2.27
Secondary	1.26	0.216	0.87	1.82
Higher	1.39	0.153	0.89	2.17
Marital Status				
Currently Married (ref)				
Living with a man	1.03	0.746	0.85	1.26
Not in union	1.01	0.917	0.81	1.26
Contraceptive use at the time of conception				
No (ref)				
Yes	1.48	**0.033**	1.03	2.14
Age at first sex				
> 18 years (ref)				
< 18 years	1.57	**< 0.001**	1.33	1.85
Parity				
No birth (ref)				
1–3	1.29	0.079	0.97	1.70
4–5	1.41	0.051	1.00	2.00
6 or more	1.19	0.435	0.77	1.82
*Community Characteristics*				
Region				
Western (ref)				
Central	0.68	**0.017**	0.49	0.93
Greater Accra	0.78	0.094	0.59	1.04
Volta	0.73	0.056	0.52	1.01
Eastern	0.75	0.059	0.56	1.01
Ashanti	0.87	0.346	0.66	1.16
Brong Ahafo	0.76	0.081	0.55	1.03
Northern	0.46	**0.019**	0.24	0.88
Upper east	0.24	**0.000**	0.12	0.50
Upper west	0.49	**0.047**	0.24	0.99
Residence				
Rural (ref)				
Urban	1.29	**0.009**	1.07	1.57
Community level of education				
Low (ref)				
High	1.00	0.973	0.81	1.24
Community media exposure				
Low (ref)				
Medium	1.13	0.224	0.93	1.38
Community knowledge of ovulation				
Low (ref)				
Medium	0.98	0.858	0.79	1.21
High	1.00	0.982	0.80	1.26
Model Detail				
Population size	4917.40			
Number of observations	3702			
Number of primary sampling units	769			
Design df	768			
F (26, 743)	6.24			
Prob.> F	0.000			
McKelvey and Zavoina’s R^2^	0.147			

## Discussion

The study examines the prevalence and factors associated with repeat induced abortions among women with a history of induced abortion in Ghana. Our results showed that the prevalence (34.7%) of repeat induced abortion in Ghana is comparable to other studies conducted in some sub-Saharan African countries; Ethiopia (34.9% and 35.4%) [1, 27] and Sudan (40%) [28]. It is also similar to other studies conducted in high-income countries; Finland (32%) [29], Canada (35.5%) [30], New Zealand (36%) [31] and Sweden (37%) [32]. However, it is higher than a study conducted in Kenya (9%) [33]. This could be due to the differences in socio-demographic characteristics of participants, cultural and legal context of the study area.

In addition, a repeat induced abortion prevalence of 56.6% was reported in China [27], which is relatively higher than this study’s prevalence. This result in China may be because abortion was part of China’s family planning services at the time of the implementation of the one-child policy. The one-child policy led to cases of sex-selective abortions because of the traditional preference for male children. Furthermore, within the context of Ghana could be attributed to the introduction of the Reducing Maternal Mortality and Morbidity (R3M) program, which aimed at not only improving access to family planning services but also comprehensive abortion care services [34].

In terms of regional differences in predicting repeat induced abortion among women, the findings showed that women residing in the Central, Northern, Upper West and East regions were less likely to practice repeat induced abortion than those from the Western region. The lower probability of women in the northern zone of Ghana practicing repeat induced abortion may be their perception of illegality, danger and public shame associated with induced abortion [35].

With regard to place of residence, the findings of the study showed that women residing in urban areas were significantly more likely to engage in repeat abortion than their rural counterparts. This result supports the findings of other studies in sub-Saharan Africa [33, 36–39]. The possible explanation for this occurrence is that urban women may have easy access to well-equipped health facilities to terminate unplanned pregnancy safely, cultural control mechanisms that prohibit pregnancy termination are more liberal in urban areas, urban women are usually found to have early premarital sex, resulting in unplanned pregnancy leading to repeat abortion, and urban women also have a higher chance of surviving a previous abortion.

The reproductive ages of women are positively associated with induced abortion practices [40]. In this study, repeated induced abortion was significantly associated with increasing age of women, which was supported by findings of previous studies in Ghana [41], and Ethiopia [1]. The plausible reason is that older women who engage in induced abortion practices during their youthful age are likely to continue the act when they advance in age compared to younger women.

Numerous studies have established a strong association between contraception and induced abortion practices among women [37, 40, 42–44] and this study was an exception. Our findings revealed that women using contraceptives at the time of conception were more likely to have engaged in repeat induced abortion relative to those who were not using contraceptive at the time of conception. This result does not commensurate with the findings of earlier studies [14, 40, 45]. The possible explanation for this phenomenon is that the women may not have gone through counselling on the use of postabortion contraception because of the threat repeat abortion poses to the health of women. Further, it could be as a result of contraceptive failure.

Early age at first sexual intercourse has a strong implication on the sexual and reproductive health of women and the number of sexual partners and affects their quality of life [46]. Our findings showed that women who had their sexual debut before attaining 18 years and above were more likely to undertake repeat induced abortion compared to women who had their first sexual intercourse after age 18. The results of this study are in line with the findings of previous studies [38, 45], where these studies found higher rates of repeated induced abortion among women who had their first before reaching the age of 18. For instance, Stone and Ingham [6] explained that the early sexual experience of women could be seen as an indicator of an increased likelihood of experiencing repeat abortion.

This study found a higher likelihood of repeat induced abortion among women who had attained middle levels of education compared to those with no formal education. While some earlier studies reported contrary results [1, 7, 36, 38], others found similar findings [33, 37, 40]. Studies that found lower rates of repeated induced abortion among low educated women argued that a low level of education is strongly associated with a lower use of contraceptives, which leads to unintended pregnancies and subsequent abortion [33]. However, other studies that found higher rates of repeated induction among educated women suggest that educated women may have financial ability and easy access to safe abortion services [40].

Overall, this study aligns with both rational choice and social learning theories positing that individual (e.g., age, education, contraceptive use, age at first sex) and community level factors (residence and region) influence the likelihood of repeat induced among study population. Based on rational choice theory, our results indicate that a woman aged 35–49 (older adults) may choose the option to have an abortion of an unintended pregnancy to lower the cost of raising another child if she already has some children. Social learning theorists would say women in urban centers would practice induced abortion if they are modelling such behaviour from their immediate social circles, consequences of induced abortion are not unpleasant enough to hinder future occurrences [21].

### Strengths and limitations of the study

The main strength of this study is the use of nationally representative data to examine the prevalence of and factors associated with repeat induced abortions among women with a history of induced abortion in Ghana. This finding therefore can be generalized to all women of reproductive age in Ghana. Regardless of this outlined strength, this was a cross-sectional study, and it will be difficult to deduce any causal interpretation. Moreover, because the study used secondary data, it could not account for other factors at the community and national levels that might have influenced repeat-induced abortion practices among women of reproductive age. The measurement of induced abortion was based on women’s self-reporting and hence what was reported could be termed as a minimum estimate. The illegality of abortion in Ghana could also pose the risk of underreporting.

## Conclusion

Abortion is considered a sensitive issue in Africa because of the cultural, moral, legal and social meanings that surround it and has implications for morbidity, mortality and fertility trends. This study identified and examined individual and community factors associated with women having repeat induced abortion in Ghana. The prevalence of repeat abortion in this study is 34.7%. We found factors associated with the likelihood of repeat induced abortion as increasing age of women, attaining middle/JHS education, using contraceptive at the time of conception, residing in urban areas and early sexual debut. On the contrary, protective factors of repeat abortion include residing in central, northern, upper west and upper east regions. Future policies and interventions should intensify sexual reproductive health education among those with low education, adult, urban society and those residing in the northern zone of Ghana.

## Data Availability

The datasets generated and/or analyzed during the current study are available in the MEASURE DHS database at repository; http://dhsprogram.com/data/available-datasets.cfm.

## Abbreviations

GMHS: Ghana Maternal Health Survey
AOR: Adjusted odds ratios
CIs: confidence intervals
JHS: Junior High School
SSA: sub-Saharan Africa
SDGs: sustainable development goals
GHS: Ghana Health Service
GSS: Ghana Statistical Service
DHS: Demographic and Health Survey
R3: M Reducing Maternal Mortality and Morbidity (R3 M) program
